# Manual Therapy in Cervical and Lumbar Radiculopathy: A Systematic Review of the Literature

**DOI:** 10.3390/ijerph18116176

**Published:** 2021-06-07

**Authors:** Tomasz Kuligowski, Anna Skrzek, Błażej Cieślik

**Affiliations:** 1 Faculty of Physiotherapy, University School of Physical Education in Wroclaw, 51-612 Wroclaw, Poland; tomasz.kuligowski@awf.wroc.pl (T.K.); anna.skrzek@awf.wroc.pl (A.S.); 2 Faculty of Health Sciences, Jan Dlugosz University in Czestochowa, 42-200 Czestochowa, Poland

**Keywords:** manual therapy, low back pain, neck pain, radiculopathy, spine

## Abstract

The aim of this study was to describe and update current knowledge of manual therapy accuracy in treating cervical and lumbar radiculopathy, to identify the limitations in current studies, and to suggest areas for future research. The study was conducted according to PRISMA guidelines for systematic reviews. A comprehensive literature review was conducted using PubMed and Web of Science databases up to April 2020. The following inclusion criteria were used: (1) presence of radiculopathy; (2) treatment defined as manual therapy (i.e., traction, manipulation, mobilization); and (3) publication defined as a Randomized Controlled Trial. The electronic literature search resulted in 473 potentially relevant articles. Finally, 27 articles were accepted: 21 on cervical (CR) and 6 in lumbar radiculopathy (LR). The mean PEDro score for CR was 6.6 (SD 1.3), and for LR 6.7 (SD 1.6). Traction-oriented techniques are the most frequently chosen treatment form for CR and are efficient in reducing pain and improving functional outcomes. In LR, each of the included publications used a different form of manual therapy, which makes it challenging to summarize knowledge in this group. Of included publications, 93% were either of moderate or low quality, which indicates that quality improvement is necessary for this type of research.

## 1. Introduction

Radiculopathy is described as nerve root irritation resulting from various pathologies, including herniated intervertebral disc (22% cases), bone spurs, spinal instability, and trauma [1,2]. Upper and lower limb pain can be referred to as the main symptom of cervical or lumbar pathology. Other symptoms usually include muscle weakness, local pain, motor, sensory, or reflex deficits [3,4].

Cervical radiculopathy (CR) is most prevalent in individuals over 40 years of age, with an annual incidence of 83.2 per 100,000 persons [5]. This makes it less common than lumbar radiculopathy (LR) [3] (also known as sciatica), whose prevalence has been documented in the USA as high as 25% of all lower back pain (LBP) cases [6] and represents the most common complaint among patients visiting a spine surgeon [7,8]. Due to its severe manifestation and the lack of treatment standardization, irrespective of healthcare system type, radiculopathy causes substantial socio-economic problems and limits daily living activities due to disability and inability to work that can last up to 20 weeks after surgical treatment [9,10,11].

Referred symptoms, including pain, cause more significant disability when compared to local pain alone [12]. Although radiculopathy remains a challenge for both researchers and clinicians, various non-operative forms of treatment are used to improve patients’ outcomes. The successful treatment method is non-surgical in 75%–90% of cases suffering from cervical radiculopathy (CR) [13,14,15]. In recent years, studies have shown the effectiveness of physical therapy involving strengthening or stretching, and also various forms of manipulative therapy for radiculopathy [1,16,17,18].

Manual therapy forms can be joint-oriented (mobilization, manipulation, traction), soft-tissue-oriented (massage forms), neural-tissue-oriented (neurodynamic), or mixed (specific exercises). Most of these treatments are successful in improving radiculopathy symptoms [19,20], but the quality of evidence might often be questioned. There is still only low-level evidence that neural mobilizations can be successful as a standalone method [21]. Little is known about joint mobilization efficacy alone in treating radiculopathy. While its biomechanical background remains unclear [22], one of the most commonly used manual therapy methods is traction, but evidence on its efficacy, whether applied alone or combined, needs further research [23,24]. While numerous CR reviews can be found in the literature in recent years [5,22,25,26,27,28,29], those regarding the lumbar region are minimal [7,9,27] and often of poor quality [30]. The latest reviews regarding CR and LR come from 2016 [5] and 2017 [30] respectively, which was encouraging.

The aim of this study was to (a) describe and update knowledge of manual therapy accuracy in treating cervical and lumbar radiculopathy; (b) to identify the limitations of current studies; and (c) to suggest areas for future research.

## 2. Materials and Methods

### 2.1. Design and Protocol

The study design was a systematic review and was conducted following the PRISMA guidelines. The protocol was registered a priori in the PROSPERO database under the following registration number: CRD42020143399.

### 2.2. Search Strategy and Selection Criteria

Publications (up to 30 April 2020) were searched in PubMed and Web of Science. Additionally, we conducted a manual search in the references of the included articles. The review included only publications in English. The following inclusion criteria were used: (a) presence of radiculopathy and/or radicular pain, and/or sciatica (for lumbosacral region); (b) treatment defined as manual therapy (commonly used term for manual forms of physical therapy including traction, manipulation, mobilization of the joints and soft tissues including fascial techniques); (c) publication defined as a Randomized Controlled Trial (RCT); and (d) English language. Studies of surgical radiculopathy treatment, or those not performing between-group analyses for the measured outcomes, were excluded from the review. The following keywords were used to search for an appropriate article: (radiculopath* OR hernia*) AND (manual therapy OR mobilization OR manipulation OR traction). Radiculopathy localization was not determined before the search; however, it was extracted at the data analysis stage. Grey literature was not searched in this review.

### 2.3. Data Extraction and Quality Assessment

A data extraction form was created to extract relevant data (publication year, study population, manual therapy intervention type, primary outcome of the study, and study conclusion). Screening of research records and risk of bias assessment was conducted by two independent reviewers (T.K. and B.C.), with the intervention of a third researcher (A.S.) in case of disagreement. Included studies underwent a methodological quality assessment for risk of bias using the Physiotherapy Evidence Database (PEDro) scale. This scale consists of a checklist of 11 scored yes-or-no questions giving a methodological quality score. Score 9 to 10 is considered excellent, 6 to 8 is good, 4 to 5 is fair, and 3 or below represents poor quality [31]. If the publication was in the PEDro database, the PEDro score was extracted. Other studies were manually evaluated. For each study, an additional internal validity score (IVS) was calculated. The PEDro scale deals with various aspects of RCT analysis, such as internal validity or external validity. Therefore, as a methodological quality assessment, van Tulder suggested the extraction of seven PEDro items (2, 3, and 5 through 9) [32]. Positive scores for each of these items were added together, giving a collective IVS score. A value of 6–7 is considered as high methodological quality, 4–5 is considered as a moderate methodological quality, and 0–3 points represent a study with limited methodological quality [32,33].

## 3. Results

### 3.1. Quality Assessment

The mean PEDro score of all included RCTs was 6.6 (SD = 1.4; range: 5–9) out of 10. For CR, the score was 6.6 (SD = 1.3; range: 5–9), and for LR the score was 6.7 (SD = 1.6; range: 5–9). Based on IVS, out of the 26 analyzed publications, three publications (11%) obtained a score classifying the quality of the publication as ‘high’, 12 (44%) as ‘moderate’, and 12 (44%) as ‘limited’. Analyzing the individual items of the PEDro questionnaire, the analyzed publications most often lost points for a failure to refer to blinding of the therapists (96%), the participants (93%), and the assessors (41%). Further points were lost for a failure to meet the criterium of ‘concealed allocation’ (44%) and ‘intention-to-treat analysis’ (30%). Table 1 presents the methodological quality of the included studies.

### 3.2. Literature Search

**Figure 1:**
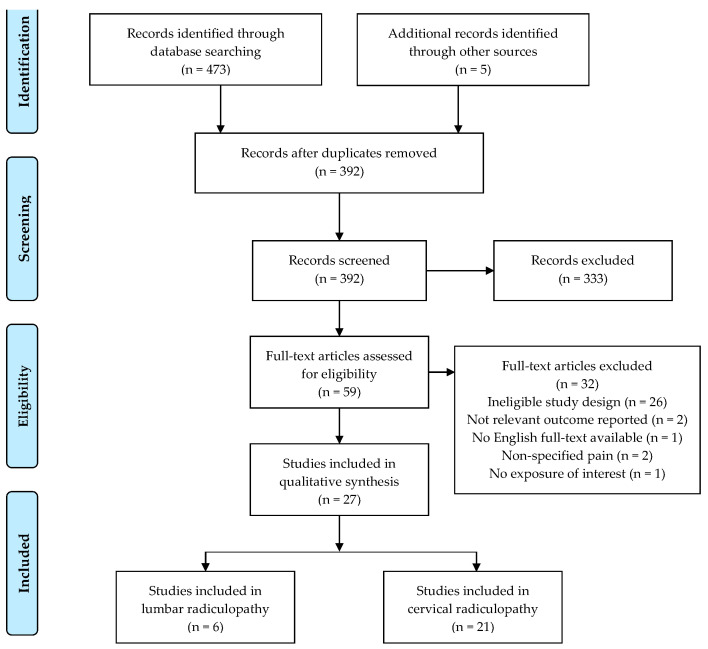
Study selection flowchart, according to PRISMA guidelines.

The electronic literature search resulted in 473 potentially relevant articles. After removing duplicate articles, 392 articles qualified for the title and abstract analysis. At this stage, 333 items were rejected, while 59 were accepted for full-text analysis. Finally, after considering the eligibility criteria for the review, 27 articles were accepted: 21 for cervical and 6 for lumbar radiculopathy. The list of excluded studies has been provided as a Supplementary File (Table S1). The most common reason for rejection was a failure to meet the inclusion criterion, i.e., unpublished work in English or study design not being RCT. Grey literature has not been included. Figure 1 illustrates a flowchart of study selection.

### 3.3. Study Characteristics

Table 2 illustrates the characteristics of the included studies. The studies included in this review used two different pain measures: Numerical Pain Rating Scale (NPRS) and Visual Analogue Scale (VAS). For CR, the most common outcome measures were the Neck Disability Index (NDI) and range of motion (ROM). Single studies used QuickDASH, grip strength, Patient-Specific Functional Scale (PSFS), Global Rating Of Change (GROC), and The Short Form Health Survey (SF-36). For LR, studies commonly used Straight Leg Raise range of motion (SLR ROM), Oswestry Disability Index (ODI), and SF-36.

### 3.4. Types of Manual Therapy

In seven studies, as an intervention, one of the studied groups received manual therapy alone (four in CR and three in LR). Most often, manual therapy was combined with exercises and physical therapy (electrotherapy, hot packs, and ultrasounds). In CR, twelve studies used cervical traction. In LR, every study used different manual therapy techniques: one study used mobilization, one traction, one manipulation, one flexion-distraction technique, and one The Mulligan bent leg raise.

## 4. Discussion

The first purpose of this study was to describe current knowledge regarding the effectiveness of manual therapy in CR and LR. Functional outcome is considered to be the main criterium in assessing an intervention’s efficacy for CR and LR, which can divide patients’ treatment into surgical or non-surgical. The most specific, with internal consistency and excellent test-retest reliability [61,62] assessment, (the NDI) has been used in most studies as a functional outcome measurement tool for CR. There was no such consistency for LR patients’ outcomes because of low specificity in radiculopathy and low evidence of one-dimensionality of ODI [63]. A reliable tool to assess patients’ self-reported outcomes for LR is unavailable.

### 4.1. Cervical Radiculopathy

Treatment with CR, unlike LR, mainly focused on traction techniques in most authors. This situation is due mainly to a much more comfortable grip and control in the cervical spine than in the lumbar spine, which is a more specific technique. While Ayub et al. (2019) combined traction with other treatment forms such as neural mobilization (passive vs. active), none of the treatment methods was found to be superior to the others [38]. Afzal et al. (2019) also compared manual traction, manual opening techniques, and a combination of these in patients with CR, but the effects of both techniques were equally effective in functional outcome [37]. Traction stood as baseline technique in many studies, and none of them showed superiority while used alone. This type of technique can be varied in specifying starting position, direction, force, amplitude, and velocity. In the gathered literature, there is a lack of detail on manual traction attributes. In most cases, this should be considered as general traction. For instance, Jellad et al. (2009) detailed it as intermittent traction, but no further information was provided [52]. Fritz et al. (2014) also used different forms of non-specific, mechanical traction combined with an exercise program that confirmed its efficacy and superiority to exercises alone, but no “traction alone” subgroup was formed [51]. Although most authors observed improvement in patients’ functional outcomes using traction or a traction component in a multimodal approach, some did not find that adding traction was successful in treating CR [53]. Shafique et al. (2019) also proved that multimodal treatment could provide better effects in patients with cervical radiculopathy [64]. This was based on spinal mobilizations, neuro-dynamics and arm movements. Cervical radiculopathy, thought to be mechanical, spatial dysfunction, also needs treatment, including movement, both proximally and distally. It has to be mentioned that a small number of papers used clinical tests for assessing functional outcomes [38,43,49,54]. This is because local pain is not the primary CR and LR problem, but distal dysfunction (e.g., muscle weakness, motor and sensory deficits due to neural malfunction), causing disability, which should always be assessed. LR also lacks in this regard, and three authors chose that way of assessing patients which, on the other hand, was more than half of all LR literature [56,57,58]. Wainner et al. (2003) proved that, for cervical radiculopathy. the ULNT tests, and especially the 1A type, are most useful for ruling out this pathology [65].

Neural mobilization is a type of technique aimed at healing neural tissue which is considered to be one of the main problems in radiculopathy after mechanical compression [55]. Nerve root will become impeded when is overstretched, or its blood supply is limited due to compression for a significantly long time, or both. Some authors applied neural mobilization techniques as a treatment for CR [38]. While Ayub et al. (2019) tried to prove the different effects comparing active and passive form of this technique in a multimodal approach, Kim et al. (2017) applied neural mobilization, different to the multimodal approach, but not using traction alone. In both cases, the effects were positive on functional outcomes [38,43], although the former author included only females, which may limit the generalizability of the results. So far, the question of neural mobilization techniques’ efficacy in CR remains unsolved.

Joint techniques are appropriate in treating joint-oriented dysfunction. This type of impairment can be taken into consideration regarding the biomechanical background of CR and LR. The relation of facet joints may be imbalanced, which can result in joint(s)’ hyper- or hypomobility. These techniques are aimed at treating hypomobile segments, while the hypermobile needs to be stabilized by in-depth muscle training. No author provides details on patients’ manual examination, called “joint play” in manual therapy, which is essential in stating whether this individual needs to be mobilized in this segment in this particular direction. Although Ayub et al. (2019) and Bukhari et al. (2016) applied mobilization in their research, it was only part of a multimodal approach aiming to differentiate traction techniques, with no further details provided on mobilized segment [38,48]. Young et al. (2019) mentioned manual therapy, but they focused mainly on thoracic spine thrust and non-thrust manipulations and unspecified neck movements without further details on a specific segment [53]. A different manipulation-oriented approach was proposed by Yang et al. (2016) based on patients’ radiographs—the group age range was high (55–75), but the effects of the manipulation were promising [66]. As well as age, inclusion criteria specified CSR (cervical spondylotic radiculopathy).

A specific exercise program has been used by several authors [48,50,51,52,53,54]. Only two authors aimed the exercise form at the biomechanical aspect of CR’s etiology, which was to increase the size of the intervertebral foramen, and no significant, positive results were observed [37,50]. Unfortunately, the authors did not provide any further details on the exercise program, besides an isometric strengthening of the muscles. Fritz et al. (2014) used a neck exercise program as a base for each of three formed groups (G1: exercise, G2: exercise + mechanical traction, G3: exercise + over-door traction) which resulted in reducing the level of neck and arm pain. The exercise program for neck included supine cranio-cervical flexion to activate deep stabilizing muscles with an air-filled pressure sensor as feedback. In contrast, scapular-strengthening exercises included prone horizontal abduction, side-lying forward flexion, prone extensions and push-ups [51]. Jellad et al. (2009) applied a “standard” rehabilitation program including ultrasound, infrared, massage, cervical spine mobilizations, and isometric muscle strengthening. No details on the above activities, such as dozing, area, direction, etc., were found, so it cannot be considered as a specific treatment method despite the fact of its efficacy in improving pain and functional outcome [52]. Young et al. (2019) proved that the the exercise program, including cervical retractions, extensions, and deep flexors’ activation, was efficient with or without adding an extra traction component. Although they described the details of every maneuver, we found no information on which specific exercise was used in each session, so it is impossible to state whether the program was consistent and repeatable [35]. Joghataei et al. (2004) used exercises including neck deep flexor strengthening as a base which showed an improvement, but significant relief was observed after adding cervical traction combined with electrotherapy [54]. Akkan et al. (2018) also proved that stabilizing exercises including of the deep neck muscles, can improve pain, quality of life and patients’ posture [67]. Wibault et al. (2017) observed promising effects using neck-specific exercises compared to the standard approach in patients who had undergone surgical treatment [68]. A similar outcome was observed by other researchers when comparing neck-specific training with a prescribed standard physical activity approach [69,70].

### 4.2. Lumbar Radiculopathy

Regarding LR, a limited number of RCTs was found to be eligible in this review. Among the five studies, few methods of treatment for LR were used by authors, and, unlike CR, no trends in choosing treatment form were observed. No unity was found in functional outcome assessment across all included studies. Only two of five studies included neurodynamic tests (SLR) [56,57]. Moustafa et al. (2013) applied a lumbar lordotic angle as an outcome, but this parameter was also an inclusion criterion [58]. Although all authors used questionnaires as an outcome, two of them decided to include only this type of examination, which makes it difficult to answer the question on individuals’ clinical improvement, as they had omitted this part.

Due to the diversity of treatment methods used, it is challenging to compare their effects. Satpute et al. (2019) applied spinal mobilizations with leg movement plus exercise and electrotherapy, compared to exercise and electrotherapy alone [56] and found significantly improved outcomes, especially in mobilization. The adjacent segments mobilization might also be helpful for LR patients and was proved by Kostadinović et al. (2020) in their studies [71]. They applied thoracic spine mobilization and lumbar stabilization. This type of approach is focused on improving hypomobile segments’ motion in the thoraco-lumbar region to reduce axial forces in lumbar segments. On the other hand, McMorland et al. (2010) compared surgical treatment (microdiscectomy) and standardized spinal manipulation by a chiropractor in patients who had not responded to other non-specific forms of non-operative treatment for at least three months. Both methods significantly improved the patient’s functional outcome and pain level. Unfortunately, no clinical examination was applied in the study, such as SLR, SLUMP, or other neurodynamic forms (e.g., EMG) [59]. Due to the different study project, joint-oriented, but with differently aimed techniques (mobilization vs. manipulation), we found it difficult to compare these two authors’ works to each other. Surgical treatment should be considered only along with the red-flag-symptoms that occurred. Another study that used the manipulation approach was that of Ghasabmahaleh et al. (2020). They observed patients’ outcomes improvements in subacute and chronic LR using Maigne’s techniques [72]. The group that underwent physiotherapy and manipulations had superior results to physiotherapy alone. Different approaches including epidural injection with manipulation were proposed by Yin et al. (2018). They observed better effects in the multimodal approach group; however, one of their methods was invasive [73].

Exercise programs are present in two out of five (40%) of our findings [56,60]. Gudavalli et al. (2006) compared the active trunk exercise program (ATEP) which is based on activation of deep, lumbar stabilizing muscles with flexion-distraction maneuver (FD). ATEP was found to be significantly more effective in the recurrent pain group with moderate to severe symptoms, while FD was better for chronic symptoms (defined by the author as pain lasting longer than three months) [60]. The first author also found the exercise program to be effective. However, the aim of the study was to prove the efficacy of a multimodal approach, rather than exercise alone [56].

When analyzing the efficacy of neural tissue mobilization, two authors applied this type of treatment [55,57]. Despite the promising conclusion of improvement in SLR and VAS outcome, Tambekar et al. (2016) did not observe a significant effect maintained in the follow-up stage [57]. The quality of this study was also limited due to the absence of concealed allocation, no blinding, no adequate follow-up, and no intention-to-treat analysis. Plaza-Manzano et al. (2019) did not find neurodynamic mobilization to be effective when combined with motor control training compared to motor control training alone [55]. However, it should be mentioned that inclusion criteria included an extensive range of participants’ age (18–60) and SLR score was considered to be eligible when the pain was reproduced only within 40–70 degrees of range.

### 4.3. Methodological Concerns

The overall quality of the included studies’ is low to moderate. Only one study designed an intervention with blind therapists [55], and two other studies designed the research with blind participants [35,50]. This is due to the specificity of treatment techniques thought to apply a biomechanical result in a specific area. In this type of intervention, blinding the therapist or physician is difficult to do, and in some cases impossible. Therefore, we treated the ‘blinding the therapist’ criterion with caution.

### 4.4. Future Directions

The main recommendations relate to the standardization of clinical examination with objective methods or specific devices and full details on the intervention. The decision-making process would be more fruitful with advanced radiological imaging and functional outcome extended by neurodynamic tests that correlate with symptoms in distal parts of the body. As symptomatic radiculopathy most often impairs the extremities’ function, it should be essential to focus on this field and control the outcome using clinical tests such as ULNTs for CR and SLR and SLUMP for LR. Insufficiently detailed information is most often found for specific techniques. No detailed pre-intervention assessment is normally provided, which complicates the selection of appropriate treatment.

### 4.5. Limitations

First, we considered only papers in English. Second, in this study, the literature review was conducted using two databases, without a grey literature search, which could limit the generalizability of obtained results. Due to the controversial homogeneity of the manual therapy methods used and the specific aim of this paper, we decided not to design our study as a meta-analysis, which could also be seen as a limitation. A small number of LR clinical trials was also a significant barrier in unifying treatment methods for this pathology. Another limitation was the poor quality of most of the available publications.

## 5. Conclusions

Traction-oriented techniques are the most frequently chosen treatment form for CR and are also efficient in reducing pain and improving functional outcomes. Mobilization techniques often lack information about the patient’s examination before the baseline, which makes it challenging to evaluate its efficacy. Exercise programs itself are efficient and improve patients’ outcomes, but there is no standardization of specific activities to specific pathology algorithm. Due to a radiculopathy background and possible symptoms, the decision-making process, including neurodynamic tests, should be mandatory for all CR and LR individuals. Based on the available literature, the multimodal approach with traction component is the most efficient for CR, and the multimodal approach with traction component, spinal mobilizations, and activation of core muscles for LR. No single-method therapy is recommended for treating both CR and LR.

## Figures and Tables

**Table 1:** Methodological quality of the included studies.

Author (Year)	(1) *	(2)	(3)	(4)	(5)	(6)	(7)	(8)	(9)	(10)	(11)	PEDro Score	IVS	Quality
Cervical radiculopathy														
Hassan et al. (2020) [34]		X		X				X		X	X	5/10	2/7	Limited
Young et al. (2019) [35]	X	X	X	X	X		X	X	X	X	X	9/10	6/7	High
Eldesoky et al. (2019) [36]	X	X	X	X				X	X	X	X	7/10	4/7	Moderate
Afzal et al. (2019) [37]	X	X					X	X	X	X	X	6/10	4/7	Moderate
Ayub et al. (2019) [38]	X	X	X	X			X	X	X	X	X	8/10	5/7	Moderate
Ojoawo and Olabode (2018) [39]	X	X		X				X	X	X	X	6/10	3/7	Limited
Song and Pan (2017) [40]	X	X	X	X			X	X	X	X		7/10	5/7	Moderate
Rodríguez-Sanz et al. (2017) [41]	X	X	X				X	X		X	X	6/10	4/7	Moderate
Cui et al. (2017) [42]		X		X				X	X	X	X	6/10	3/7	Limited
Kim et al. (2017) [43]	X	X		X				X		X	X	5/10	2/7	Limited
Khan et al. (2017) [44]	X	X		X			X	X	X	X	X	7/10	4/7	Moderate
Savva et al. (2016) [45]		X	X				X	X	X	X	X	7/10	5/7	Moderate
Khan et al. (2016) [46]	X	X		X				X	X	X		5/10	3/7	Limited
Waqas et al. (2016) [47]		X						X	X	X	X	5/10	3/7	Limited
Bukhari et al. (2016) [48]	X	X		X				X		X	X	5/10	2/7	Limited
Costello et al. (2016) [49]	X	X	X	X			X	X	X	X	X	8/10	5/7	Moderate
Langevin et al. (2015) [50]	X	X	X	X	X		X	X	X	X	X	9/10	6/7	High
Fritz et al. (2014) [51]	X	X	X	X			X	X	X	X	X	8/10	5/7	Moderate
Jellad et al. (2009) [52]	X	X		X			X	X		X	X	6/10	3/7	Limited
Young et al. (2009) [53]	X	X	X	X			X	X	X	X	X	8/10	5/7	Moderate
Joghataei et al. (2004) [54]	X	X		X			X	X		X	X	6/10	3/7	Limited
Lumbar radiculopathy														
Plaza-Manzano et al. (2019) [55]	X	X	X	X		X	X	X	X	X	X	9/10	6/7	High
Satpute et al. (2018) [56]	X	X	X	X			X	X	X	X	X	8/10	5/7	Moderate
Tambekar et al. (2015) [57]	X	X		X			X			X	X	5/10	2/7	Limited
Moustafa et al. (2013) [58]	X	X	X	X				X	X	X	X	7/10	4/7	Moderate
McMorland et al. (2010) [59]	X	X	X	X					X	X	X	6/10	3/7	Limited
Gudavalli et al. (2006) [60]	X	X	X	X						X	X	5/10	2/7	Limited
%, X	85	100	56	85	7	4	59	89	70	100	93			

(1) Eligibility criteria; (2) Random allocation; (3) Concealed allocation; (4) Baseline comparability; (5) Blind participants; (6) Blind therapists; (7) Blind assessors; (8) Adequate follow-up; (9) Intention-to-treat analysis; (10) Between-group comparisons; (11) Point estimates and variability; * Eligibility criteria item does not contribute to total Physiotherapy Evidence Database (PEDro) score; IVS: internal validity score.

**Table 2:** Characteristics of included studies.

Author (Year)	Groups Characteristic (Mean Age, Sex)	*n*	Interventions	Outcome Measures	Conclusions
Cervical radiculopathy					
Hassan et al. (2020) [34]	G1: 43.0 (14M, 6F)	G1: 20	G1: Kaltenborn sustained stretch mobilization, TENS, hot packs	NPRS NDI ROM	Both oscillatory and sustained stretch mobilization techniques are found to be effective in the management of cervical radiculopathy in terms of pain, range and disability. However, oscillatory mobilization is found to be superior in terms of functional ability and range of motion.
	G2: 43.0 (13M, 7F)	G2: 20	G2: Maitland oscillatory mobilization, TENS, hot packs		
Young et al. (2019) [35]	G1: 48.8 (5M, 17F) G2: 43.1 (9M, 12F)	G1: 22 G2: 21	G1: Thoracic spine manipulation G2: Sham thoracic spine manipulation	NPRS NDI ROM	One session of thoracic manipulation resulted in improvements in pain, disability, cervical ROM, and deep neck flexor endurance in patients with cervical radiculopathy.
Eldesoky et al. (2019) [36]	G1: 43.1 (13M, F12) G2: 43.9 (14M, 11F)	G1: 25 G2: 25	G1: Maitland postero-anterior and rotation oscillatory mobilization techniques G2: Therapeutic ultrasonic and exercise program	VAS NDI Somatosensory evoked potentials	Cervical mobilization could be utilized as an effective physical therapy program design for patients with cervical radiculopathy for improvement of pain level, functional disability and nerve root function.
Afzal et al. (2019) [37]	G1: 42.1 (M, F) G2: 40.9 (M, F) G3: 42.5 (M, F)	G1: 13 G2: 13 G3: 14	G1: Opening of intervertebral foramen technique G2: Manual cervical traction G3: Combined both above techniques	NPRS NDI PSFS Active extension/extension Right/left side bending Right/left Rotation	Manual intervertebral foramen opening technique, manual traction, and combination of both techniques were equally effective in decreasing pain, level of disability and improved cervical mobility in patients with cervical radiculopathy.
Ayub et al. (2019) [38]	G1: 21.9 (0M, 22F) G2: 23.1 (0M, 22F)	G1: 22 G2: 22	G1: Cervical traction, Unilateral Posterior Anterior glide and passive upper extremity neural mobilization G2: Cervical traction, Unilateral Posterior Anterior glide and active upper extremity neural mobilization	NPRS NDI ROM	Both active and passive neural mobilization is effective in the management of cervical radiculopathy. One of the interventions is not superior to the other.
Ojoawo and Olabode (2018) [39]	G1: 51.4 (14M, 11F) G2: 55.7 (15M, 10F) G3: 59.5 (11M, 14F)	G1: 25 G2: 25 G3: 25	G1: Cervical traction plus Exercise, massage, ice therapy G2: Transverse oscillatory pressure plus Exercise, massage, ice therapy G3: Exercise, massage, ice therapy only	VAS NDI	Transverse oscillatory pressure reduces the PI and disability of patients with cervical radiculopathy more quickly, compared to conventional therapy.
Song and Pan (2017) [40]	G1: 42.4 (7M, 12F) G2: 42.5 (7M, 13F) G3: 42.2 (8M, 12F)	G1: 19 G2: 20 G3: 20	G1: Warm needling moxibustion G2: Warm needling moxibustion and Mulligan dynamic joint mobilization G3: Warm needling moxibustion and cervical traction	ROM VAS	Warm needling moxibustion plus Mulligan dynamic joint mobilization can effectively improve neck ROM and relieve pain in patients with cervical radiculopathy.
Rodríguez-Sanz et al. (2017) [41]	G1: 33.3 (14M, 11F) G2: 32.5 (12M, 15F)	G1: 25 G2: 27	G1: Cervical lateral glide G2: Waiting list (without intervention)	NPRS QuickDASH Ipsilateral cervical rotation	Cervical lateral glide is superior to the absence of treatment in reducing pain and increasing the affected upper limb function of participants who suffer from cervicobrachial pain.
Cui et al. (2017) [42]	G1: 44.1 (45M, 128F) G2: 44.4 (35M, 141F)	G1: 173 G2: 176	G1: Shi-style cervical manipulations G2: Mechanical cervical traction	NDI VAS SF-36	Shi-style cervical manipulations could be a better option than mechanical cervical traction for the treatment of cervical radiculopathy-related pain and disability.
Kim et al. (2017) [43]	G1: 29.3 (5M, 10F) G2: 29.3 (6M, 9F)	G1: 15 G2: 15	G1: Manual cervical traction G2: Manual cervical traction and neural mobilization	NPRS NDI ROM Cranio-Cervical Flexion Test	These results suggest that the neural mobilization can contribute to pain relief, recovery from neck disability, ROM, and deep flexor endurance for patients with cervical radiculopathy.
Khan et al. (2017) [44]	G1: 43.1 (16M, 4F) G2: 48.8 (16M, 4F)	G1: 20 G2: 20	G1: Intermittent cervical traction in sitting position, TENS, hot pack G2: Intermittent cervical traction in supine position, TENS, hot pack	NDI	Supine position is a better choice for applying cervical traction as compared to sitting position for the management of cervical radiculopathy when comparing post interventional NDI score
Savva et al. (2016) [45]	G1: 45.2 (8M, 13F) G2: 49.2 (8M, 13F)	G1: 21 G2: 21	G1: Neural mobilization and intermittent cervical traction G2: Participants did not receive any type of treatment	NPRS PSFS NDI Grip strength ROM	Neural mobilization with simultaneous intermittent cervical traction can improve pain, function, disability, grip strength and cervical range of motion in people with cervical radiculopathy.
Khan et al. (2016) [46]	G1: 38.0 (25M, 25F) G2: 38.0 (25M, 25F)	G1: 50 G2: 50	G1: Manual cervical traction and a combination of conventional exercises and modalities including TENS and superficial thermotherapy. G2: A combination of conventional exercises and modalities including TENS and superficial thermotherapy.	VAS	Manual cervical traction when used with conventional exercises and modalities was an effective method for decreasing pain in cervical radiculopathy.
Waqas et al. (2016) [47]	G1: 47.0 (29M, 21F) G2: 47.0 (34M, 16F)	G1: 50 G2: 50	G1: Maitland Thoracic spine manipulation G2: Maitland cervical spine mobilization	NPRS NDI	The result shows that Maitland Thoracic spine manipulation and Maitland cervical spine mobilization were effective techniques for pain reduction and functional abilities restoration.
Bukhari et al. (2016) [48]	G1: Not specified G2: Not specified	G1: 21 G2: 15	G1: Segmental mobilization and exercise therapy and manual traction G2: Segmental mobilization and exercise therapy and mechanical traction	NPRS NDI	If cervical radiculopathy patients are treated with mechanical traction, segmental mobilization, and exercise therapy, pain and disability will be managed more effectively than when treated with manual traction, segmental mobilization, and exercise therapy.
Costello et al. (2016) [49]	G1: 46.2 (sex not specified) G2: 42.0 (sex not specified)	G1: 12 G2: 11	G1: Soft tissue mobilization G2: Therapeutic Ultrasound	NDI GROC PSFS NPRS ROM	Patients with neck and arm pain demonstrated greater improvements in ROM, GROC, and PSFS, and pain following soft tissue mobilization than after receiving therapeutic ultrasounds.
Langevin et al. (2015) [50]	G1: 42.8 (6M, 12F) G2: 47.8 (6M, 12F)	G1: 18 G2: 18	G1: Manual therapy and exercise program aimed at increasing the size of the intervertebral foramen G2: Manual therapy and exercise program without the specific goal of increasing the size of the intervertebral foramen	NDI QuickDASH NPRS	Results suggest that manual therapy and exercises are effective in reducing pain and functional limitations related to CR. The addition of techniques thought to increase the size of the intervertebral foramen of the affected nerve root yielded no significant additional benefits.
Fritz et al. (2014) [51]	G1: 44.9 (10M, 18F) G2: 48.1 (18M,13F) G3: 47.6 (12M, 15F)	G1: 28 G2: 31 G3: 27	G1: Exercise alone G2: Exercise and mechanical traction G3: Exercise and over-door traction	NDI VAS	Adding mechanical traction to exercise for patients with cervical radiculopathy resulted in lower disability and pain, particularly at long-term follow-ups.
Jellad et al. (2009) [52]	G1: 38.5 (4M, 9F) G2: 44.2 (3M,10F) G3: 41.3 (2M, 11F)	G1: 13 G2: 13 G3: 13	G1: Conventional rehabilitation with intermittent manual traction G2: Conventional rehabilitation with intermittent mechanical traction G3: Conventional rehabilitation alone	VAS	Manual or mechanical cervical traction appears to be a major contribution in the rehabilitation of cervical radiculopathy particularly if it is included in a multimodal approach to rehabilitation.
Young et al. (2009) [53]	G1: 47.8 (14M, 31F) G2: 46.2 (12M, 24F)	G1: 45 G2: 36	G1: Manual therapy, exercise, and intermittent cervical traction G2: Manual therapy, exercise, and sham intermittent cervical traction	NDI NPRS PSFS	The results suggest that the addition of mechanical cervical traction to a multimodal treatment program of manual therapy and exercise yields no significant additional benefit to pain, function, or disability in patients with cervical radiculopathy.
Joghataei et al. (2004) [54]	G1: 47.5 (8M, 7F) G2: 46.3 (7M, 8F)	G1: 15 G2: 15	G1: Cervical traction and electrotherapy/exercise G2: Electrotherapy/exercise treatment	Grip strength	The application of cervical traction combined with electrotherapy and exercise produced an immediate improvement in the hand grip function in patients with cervical radiculopathy.
Lumbar radiculopathy					
Plaza-Manzano et al. (2019) [55]	G1: 47.0 (8M,8F) G2: 45.5 (8M, 8F)	G1: 16 G2: 16	G1: Neurodynamic mobilization plus motor control exercises G2: Motor control exercises	NPRS PLE PPT RMQ	The addition of neurodynamic mobilization to a motor control exercise program led to reductions in neuropathic symptoms and mechanical sensitivity, but did not result in greater changes of pain.
Satpute et al. (2018) [56]	G1: 49.9 (14M, 16F) G2: 42.3 (20M, 10F)	G1: 30 G2: 30	G1: Spinal mobilization with leg movement, exercise and electrotherapy G2: Exercise and electrotherapy alone	VAS ODI GROC SLR ROM	In patients with lumbar radiculopathy, the addition of spinal mobilization with leg movement, exercise and electrotherapy provided significantly improved benefits in leg and back pain, disability, SLR ROM, and patient satisfaction in the short and long term.
Tambekar et al. (2015) [57]	G1: 34.1 (8M, 8F) G2: 32.3 (7M, 8F)	G1: 16 G2: 15	G1: Mulligan bent leg raise G2: Butler’s neural tissue mobilization	VAS SLR ROM	The study showed that both techniques produce immediate improvement in pain and SLR range, but this effect was not maintained during the follow up period.
Moustafa et al. (2013) [58]	G1: 43.9 (19M, 13F) G2: 43.2 (17M, 15F)	G1: 32 G2: 32	G1: Lumbar extension traction in addition to hot packs and interferential therapy G2: Hot packs and interferential therapy	Lumbar lordotic angle NPRS ODI Modified Schober test EMG	The traction group receiving lumbar extension traction in addition to hot packs and interferential therapy experienced better effects than the control group with regard to pain, disability, H-reflex parameters and segmental intervertebral movements.
McMorland et al. (2010) [59]	G1: 41.5 (6M, 7F) G2: 42.4 (2M, 9F)	G1: 13 G2: 11	G1: Microdiscectomy G2: Spinal manipulation	MGP RMQ SF-36	Sixty percent of patients with sciatica who had failed other medical management benefited from spinal manipulation to the same degree as if they underwent surgical intervention. Of 40% left unsatisfied, subsequent surgical intervention confers excellent outcome. Patients with symptomatic LDH failing medical management should consider spinal manipulation followed by surgery if warranted.
Gudavalli et al. (2006) [60]	G1: 42.2 (81M, 42F) G2: 40.9 (66M, 46F)	G1: 123 G2: 112	G1: Flexion-distraction G2: Active trunk exercise program	VAS RMQ SF-36	Subgroup analysis indicated that subjects categorized as chronic, with moderate to severe symptoms, and those with radiculopathy, improved most with flexion-distraction. Subjects categorized with recurrent pain and moderate to severe symptoms improved most with an active trunk exercise program.

G1: group 1; G2: group 2; G3: group 3; VAS: Visual Analogue Scale; ODI: Oswestry Disability Index; GROC: Global Rating Of Change; SLR: Straight Leg Raise; ROM: Range Of Motion; RMQ: Roland Morris Questionnaire; MGP: Mcgill Pain Questionnaire; SF-36: The Short Form Health Survey; NPRS: Numerical Pain Rating Scale; NDI: Neck Disability Index; PSFS: Patient-Specific Functional Scale; GRC: Global Rating Of Change; PPT: pressure pain threshold.

## Data Availability

The data that support the findings of this study are available from the corresponding author, upon reasonable request.

## Supplementary Materials

The following are available online at https://www.mdpi.com/article/10.3390/ijerph18116176/s1. Table S1: List of excluded studies during full text assessment along with reasons for exclusion.
