# Supplementary material for: Manual Therapy in Cervical and Lumbar Radiculopathy: A Systematic Review of the Literature
Source: Int J Environ Res Public Health. 2021 Jun 7;18(11):6176. doi: 10.3390/ijerph18116176 (PMC8201115; doi:10.3390/ijerph18116176)
Supplement: Supplementary file 1 [file ijerph-18-06176-s001.zip › ijerph-1196039-supplementary.pdf]

S1 Table: List of excluded studies during full text assessment along with reasons for exclusion

Full-text articles excluded, with reasons (n=32)

Ineligible study design: 26
Not relevant outcome reported: 2
No English full-text available:1
Non-specified pain: 2
No exposure of interest: 1

Study	Reason for exclusion
Thalhamer et al. (2019) [1]	Ineligible study design
Dugailly et al. (2018) [2]	Ineligible study design
Wibault et al. (2017) [3]	Not relevant outcome reported
Kjaer et al. (2017) [4]	Ineligible study design
Stochkendahl et al. (2018) [5]	Ineligible study design
Clijsters et al. (2014) [6]	Ineligible study design
Anandkumar et al. (2015) [7]	Ineligible study design
Basson et al. (2014) [8]	Ineligible study design
Burns et al. (2011) [9]	Ineligible study design
Colloca et al. (2004) [10]	Ineligible study design
Hanten et al. (2000) [11]	Not relevant outcome reported
Pennetti (2018) [12]	Ineligible study design
Romeo et al. (2018) [13]	Ineligible study design
Andersen et al. (2017) [14]	No English full-text available
Deschenes et al. (2017) [15]	Ineligible study design
Beltran-Alacreu et al. (2015) [16]	Non-specified pain
Childress et al. (2016) [17]	Ineligible study design
Ridehalgh et al. (2016) [18]	Ineligible study design
Thoomes et al. (2016) [19]	Ineligible study design
Yang et al. (2016) [20]	Ineligible study design
Zhu et al. (2016) [21]	Ineligible study design
Ali et al. (2014) [22]	Non-specified pain
Efstathiou et al. (2015) [23]	Ineligible study design
Han et al. (2015) [24]	Ineligible study design
Savva et al. (2013) [25]	Ineligible study design
Rodine et al. (2012) [26]	Ineligible study design
Boyles et al. (2011) [27]	Ineligible study design
Leininger et al. (2011) [28]	Ineligible study design
Salom-Moreno et al. (2014) [29]	No exposure of interest
Cleland et al. (2007) [30]	Ineligible study design
Basson et al. (2015) [31]	Ineligible study design
Francio et al. (2017) [32]	Ineligible study design

References of excluded studies

1. Thalhamer, C.; Hahne, J.; Matthijs, O.; Machacek, P. Inter-Rater Reliability of Pain Provocation Tests for Painful Lumbar Facet Joints. A Pilot Study. *Z Orthop Unfall* **2019**, *157*, 254–262, doi:10.1055/a-0748-6081.

2. Dugailly, P.-M.; Beyer, B.; Salem, W.; Feipel, V. Morphometric Changes of the Cervical Intervertebral Foramen: A Comparative Analysis of Pre-Manipulative Positioning and Physiological Axial Rotation. *Musculoskelet Sci Pract* **2018**, *34*, 97–102, doi:10.1016/j.msksp.2018.01.007.

3. Wibault, J.; Öberg, B.; Dedering, Å.; Löfgren, H.; Zsigmond, P.; Persson, L.; Andell, M.; R Jonsson, M.; Peolsson, A. Neck-Related Physical Function, Self-Efficacy, and Coping Strategies in Patients With Cervical Radiculopathy: A Randomized Clinical Trial of Postoperative Physiotherapy. *J Manipulative Physiol Ther* **2017**, *40*, 330–339, doi:10.1016/j.jmpt.2017.02.012.

4. Kjaer, P.; Kongsted, A.; Hartvigsen, J.; Isenberg-Jørgensen, A.; Schiøttz-Christensen, B.; Søborg, B.; Krog, C.; Møller, C.M.; Halling, C.M.B.; Lauridsen, H.H.; et al. National Clinical Guidelines for Non-Surgical Treatment of Patients with Recent Onset Neck Pain or Cervical Radiculopathy. *Eur Spine J* **2017**, *26*, 2242–2257, doi:10.1007/s00586-017-5121-8.

5. Stochkendahl, M.J.; Kjaer, P.; Hartvigsen, J.; Kongsted, A.; Aaboe, J.; Andersen, M.; Andersen, M.Ø.; Fournier, G.; Højgaard, B.; Jensen, M.B.; et al. National Clinical Guidelines for Non-Surgical Treatment of Patients with Recent Onset Low Back Pain or Lumbar Radiculopathy. *Eur Spine J* **2018**, *27*, 60–75, doi:10.1007/s00586-017-5099-2.

6. Clijsters, M.; Fronzoni, F.; Jenkins, H. Chiropractic Treatment Approaches for Spinal Musculoskeletal Conditions: A Cross-Sectional Survey. *Chiropr Man Therap* **2014**, *22*, 33, doi:10.1186/s12998-014-0033-8.

7. Anandkumar, S. The Effect of Sustained Natural Apophyseal Glide (SNAG) Combined with Neurodynamics in the Management of a Patient with Cervical Radiculopathy: A Case Report. *Physiother Theory Pract* **2015**, *31*, 140–145, doi:10.3109/09593985.2014.971922.

8. Basson, C.A.; Stewart, A.; Mudzi, W. The Effect of Neural Mobilisation on Cervico-Brachial Pain: Design of a Randomised Controlled Trial. *BMC Musculoskelet Disord* **2014**, *15*, 419, doi:10.1186/1471-2474-15-419.

9. Burns, S.A.; Mintken, P.E.; Austin, G.P.; Cleland, J. Short-Term Response of Hip Mobilizations and Exercise in Individuals with Chronic Low Back Pain: A Case Series. *J Man Manip Ther* **2011**, *19*, 100–107, doi:10.1179/2042618610Y.0000000007.

10. Colloca, C.J.; Keller, T.S.; Gunzburg, R. Biomechanical and Neurophysiological Responses to Spinal Manipulation in Patients with Lumbar Radiculopathy. *J Manipulative Physiol Ther* **2004**, *27*, 1–15, doi:10.1016/j.jmpt.2003.11.021.

11. Hanten, W.P.; Olson, S.L.; Butts, N.L.; Nowicki, A.L. Effectiveness of a Home Program of Ischemic Pressure Followed by Sustained Stretch for Treatment of Myofascial Trigger Points. *Phys Ther* **2000**, *80*, 997–1003.

12. Pennetti, A. A Multimodal Physical Therapy Approach Utilizing the Maitland Concept in the Management of a Patient with Cervical and Lumbar Radiculitis and Ehlers-Danlos Syndrome-Hypermobility Type: A Case Report. *Physiother Theory Pract* **2018**, *34*, 559–568, doi:10.1080/09593985.2017.1422207.

13. Romeo, A.; Vanti, C.; Boldrini, V.; Ruggeri, M.; Guccione, A.A.; Pillastrini, P.; Bertozzi, L. Cervical Radiculopathy: Effectiveness of Adding Traction to Physical Therapy-A Systematic Review and Meta-Analysis of Randomized Controlled Trials. *Phys Ther* **2018**, *98*, 231–242, doi:10.1093/physth/pzy001.

14. Andersen, M.Ø.; Andresen, A.K.; Lorenzen, M.D.; Isenberg-Jørgensen, A.; Støttrup, C. [Non-surgical treatment of lumbar radiculopathy]. *Ugeskr Laeger* **2017**, *179*, V05170397.

15. Deschenes, B.K.; Zafereo, J. Immediate and Lasting Effects of a Thoracic Spine Manipulation in a Patient with Signs of Cervical Radiculopathy and Upper Extremity Hyperalgesia: A Case Report. *Physiother Theory Pract* **2017**, *33*, 82–88, doi:10.1080/09593985.2016.1247307.

16. Beltran-Alacreu, H.; López-de-Uralde-Villanueva, I.; Fernández-Carnero, J.; La Touche, R. Manual Therapy, Therapeutic Patient Education, and Therapeutic Exercise, an Effective Multimodal Treatment of Nonspecific Chronic Neck Pain: A Randomized Controlled Trial. *Am J Phys Med Rehabil* **2015**, *94*, 887–897, doi:10.1097/PHM.0000000000000293.

17. Childress, M.A.; Becker, B.A. Nonoperative Management of Cervical Radiculopathy. *Am Fam Physician* **2016**, *93*, 746–754.

18. Ridehalgh, C.; Moore, A.; Hough, A. The Short Term Effects of Straight Leg Raise Neurodynamic Treatment on Pressure Pain and Vibration Thresholds in Individuals with Spinally Referred Leg Pain. *Man Ther* **2016**, *23*, 40–47, doi:10.1016/j.math.2015.12.013.

19. Thoomes, E.J. Effectiveness of Manual Therapy for Cervical Radiculopathy, a Review. *Chiropr Man Therap* **2016**, *24*, 45, doi:10.1186/s12998-016-0126-7.

20. Yang, F.; Li, W.; Liu, Z.; Liu, L. Balance Chiropractic Therapy for Cervical Spondylotic Radiculopathy: Study Protocol for a Randomized Controlled Trial. *Trials* **2016**, *17*, 513, doi:10.1186/s13063-016-1644-2.

21. Zhu, L.; Wei, X.; Wang, S. Does Cervical Spine Manipulation Reduce Pain in People with Degenerative Cervical Radiculopathy? A Systematic Review of the Evidence, and a Meta-Analysis. *Clin Rehabil* **2016**, *30*, 145–155, doi:10.1177/0269215515570382.

22. Ali, A.; Shakil-Ur-Rehman, S.; Sibtain, F. The Efficacy of Sustained Natural Apophyseal Glides with and without Isometric Exercise Training in Non-Specific Neck Pain. *Pak J Med Sci* **2014**, *30*, 872–874.

23. Efstathiou, M.A.; Stefanakis, M.; Savva, C.; Giakas, G. Effectiveness of Neural Mobilization in Patients with Spinal Radiculopathy: A Critical Review. *J Bodyw Mov Ther* **2015**, *19*, 205–212, doi:10.1016/j.jbmt.2014.08.006.

24. Han, L.; Zhao, P.; Guo, W.; Wei, J.; Wang, F.; Fan, Y.; Li, Y.; Min, Y. Short-Term Study on Risk-Benefit Outcomes of Two Spinal Manipulative Therapies in the Treatment of Acute Radiculopathy Caused by Lumbar Disc Herniation: Study Protocol for a Randomized Controlled Trial. *Trials* **2015**, *16*, doi:10.1186/s13063-015-0634-0.

25. Savva, C.; Giakas, G. The Effect of Cervical Traction Combined with Neural Mobilization on Pain and Disability in Cervical Radiculopathy. A Case Report. *Man Ther* **2013**, *18*, 443–446, doi:10.1016/j.math.2012.06.012.

26. Rodine, R.J.; Vernon, H. Cervical Radiculopathy: A Systematic Review on Treatment by Spinal Manipulation and Measurement with the Neck Disability Index. *J Can Chiropr Assoc* **2012**, *56*, 18–28.

27. Boyles, R.; Toy, P.; Mellon, J.; Hayes, M.; Hammer, B. Effectiveness of Manual Physical Therapy in the Treatment of Cervical Radiculopathy: A Systematic Review. *J Man Manip Ther* **2011**, *19*, 135–142, doi:10.1179/2042618611Y.0000000011.

28. Leininger, B.; Bronfort, G.; Evans, R.; Reiter, T. Spinal Manipulation or Mobilization for Radiculopathy: A Systematic Review. *Phys Med Rehabil Clin N Am* **2011**, *22*, 105–125, doi:10.1016/j.pmr.2010.11.002.

29. Salom-Moreno, J.; Ortega-Santiago, R.; Cleland, J.A.; Palacios-Ceña, M.; Truyols-Domínguez, S.; Fernández-de-las-Peñas, C. Immediate Changes in Neck Pain Intensity and Widespread Pressure Pain Sensitivity in Patients with Bilateral Chronic Mechanical Neck Pain: A Randomized Controlled Trial of Thoracic Thrust Manipulation vs Non-Thrust Mobilization. *J Manipulative Physiol Ther* **2014**, *37*, 312–319, doi:10.1016/j.jmpt.2014.03.003.

30. Cleland, J.A.; Fritz, J.M.; Whitman, J.M.; Heath, R. Predictors of Short-Term Outcome in People with a Clinical Diagnosis of Cervical Radiculopathy. *Phys Ther* **2007**, *87*, 1619–1632, doi:10.2522/ptj.20060287.

31. Basson, A.; Olivier, B.; Ellis, R.; Coppieters, M.; Stewart, A.; Mudzi, W. The Effectiveness of Neural Mobilizations in the Treatment of Musculoskeletal Conditions: A Systematic Review Protocol. *JBI Database System Rev Implement Rep* **2015**, *13*, 65–75, doi:10.11124/jbisrir-2015-1401.

32. Tieppo Francio, V.; Towery, C.; Davani, S.; Brown, T. Spinal Manipulation and Therapeutic Exercises in Treating Post-Surgical Resurgent Lumbar Radiculopathy. *Oxford Medical Case Reports* **2017**, *2017*, doi:10.1093/omcr/omx062.
